# Molecular Simulation of Oncostatin M and Receptor (OSM–OSMR) Interaction as a Potential Therapeutic Target for Inflammatory Bowel Disease

**DOI:** 10.3389/fmolb.2020.00029

**Published:** 2020-03-04

**Authors:** Qingqing Du, Yan Qian, Weiwei Xue

**Affiliations:** ^1^Department of Pharmacy, The Second Affiliated Hospital of Chongqing Medical University, Chongqing, China; ^2^School of Pharmaceutical Sciences, Chongqing Key Laboratory of Natural Product Synthesis and Drug Research, Chongqing University, Chongqing, China

**Keywords:** inflammatory bowel disease, oncostatin M and oncostatin M Receptor, protein-protein docking, molecular dynamics simulation, binding sites prediction

## Abstract

Therapeutics targeting cytokines such as the oncostatin M (OSM)-mediated inflammation represent a potential strategy for the treatment of inflammatory bowel disease (IBD). Despite the investigation of the specific role of the interactions between OSM and the receptor (OSMR) in IBD pathogenesis, the 3D structure of the OSM–OSMR complex remains elusive. In this work, the interaction mode between OSM and OSMR at atomic level was predicted by computational simulation approach. The interaction domain of the OSMR was built with the homology modeling method. The near-native structure of the OSM–OSMR complex was obtained by docking, and long-time scale molecular dynamics (MD) simulation in an explicit solvent was further performed to sample the conformations when OSM binds to the OSMR. After getting the equilibrated states of the simulation system, per-residue energy contribution was calculated to characterize the important residues for the OSM–OSMR complex formation. Based on these important residues, eight residues (OSM: Arg100, Leu103, Phe160, and Gln161; OSMR: Tyr214, Ser223, Asp262, and Trp267) were identified as the “hot spots” through computational alanine mutagenesis analysis and verified by additional MD simulation of R100A (one of the identified “hotspots”) mutant. Moreover, six cavities were detected at the OSM–OSMR interface through the FTMap analysis, and they were suggested as important binding sites. The predicted 3D structure of the OSM–OSMR complex and the identified “hot spots” constituting the core of the binding interface provide helpful information in understanding the OSM–OSMR interactions, and the detected sites serve as promising targets in designing small molecules to block the interactions.

## Introduction

Inflammatory bowel diseases (IBDs) are complex chronic inflammatory conditions of the gastrointestinal tract that are driven by perturbed signal pathways of cytokines such as tumor necrosis factor (TNF)-α and IL-6 (Neurath, 2014). Nowadays, anti-TNF antibodies (such as infliximab and golimumab) are mainstay therapies for IBD (Choi et al., 2017). However, there are still more than 40% of patients who are non-responsive to anti-TNF agents, making the discovery of alternative therapeutic targets a priority (Kim et al., 2017). One of those potential targets, oncostatin M (OSM)-mediated inflammation, has gained a lot of interest (Verstockt et al., 2019). It is found that high pretreatment expression of OSM is strongly associated with failure of anti-TNF therapy of patients with IBD, which revealed the role of the receptor (OSMR) as part of a unique pathway that contributes to the chronicity of intestinal inflammation (West et al., 2017).

**Figure 1:**
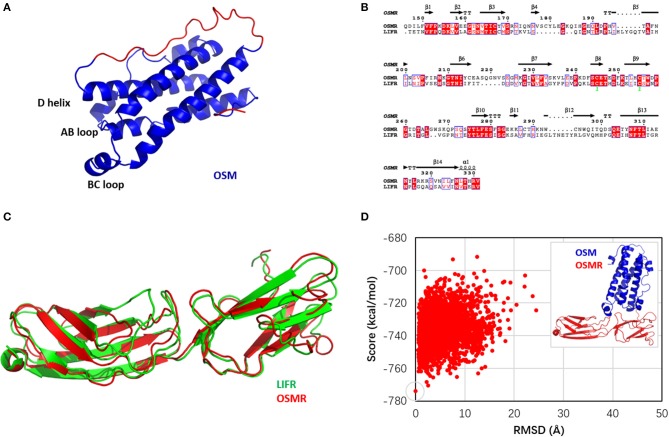
**(A)** Structure of oncostatin M (OSM); the modeled fragments are colored in red. **(B)** Sequence alignment between oncostatin M receptor (OSMR) and leukemia inhibitory factor receptor (LIFR). **(C)** Structural alignment of OSMR homology model (red) and LIFR crystal structure (green). **(D)** Docking funnel of OSM and OSMR. Inset: the top scoring conformation as near-native OSM–OSMR structure.

OSM belongs to the IL-6 family, and the activation of the OSM signal pathway is highly determined by the high affinity of OSM to the receptor (OSMR) (Adrian-Segarra et al., 2018a,b). The crystal structure of OSM reveals that the protein comprises four α helices ranging from 15 to 22 amino acids in length (termed A, B, C, and D) and linked by polypeptide loops (Figure 1A) (Deller et al., 2000). The OSMR is a member of the IL-6 receptor family that transduces signaling events of OSM (Yu et al., 2019). Currently, available antibodies, such as GSK315234 and GSK2330811, have already been proven to affect the OSM signal (Verstockt et al., 2019). Although neutralizing OSM antibodies are being developed and should be considered as a novel proof-of-concept trial in IBD patients (West et al., 2017), these developed biological medicines are large, complex, and relatively fragile molecules, which make them difficult and expensive to produce and administer on a large scale (Monaco et al., 2015).

In recent years, development of small molecule modulators targeting protein–protein interactions (PPIs) has emerged as a promising therapeutic intervention in complex diseases (Nero et al., 2014; Nim et al., 2016; Weng et al., 2019). In selecting biologically relevant protein–protein interfaces, the availability of computer-aided drug design (CADD) approach has led to the discovery of small molecules either stabilizing or disrupting the biological processes (Arkin et al., 2014; Laraia et al., 2015). The critical role for OSM in antipathogen immunity has not been described, and targeting OSM–OSMR may offer inhibition of the inflammatory pathology while preserving protective immunity (Verstockt et al., 2019). These hypotheses stimulate the idea of identification of small molecular inhibitors against the OSM–OSMR interface, which might provide safer and more broadly effective alternatives to conventional antibodies targeting monomeric macromolecules. To discover ligands specifically disrupting the OSM–OSMR interface, the information of the protein–protein interactions is needed. Unfortunately, the 3D structure of the OSM–OSMR complex remains elusive (Kim et al., 2017). It is of paramount importance to understand the details of the OSM and OSMR complex formation as well as the potential binding site between the protein–protein interface.

In this work, molecular simulation approaches aimed at filling the aforementioned gap were performed to accelerate the discovery of small molecules targeting OSM–OSMR. Starting from the crystal structure of OSM (Deller et al., 2000) and the model of the OSMR [a protein-binding region was built using the leukemia inhibitory factor receptor (LIFR) crystal structure (Huyton et al., 2007) as a template], the near-native conformation of the OSM–OSMR complex was obtained through protein–protein docking. The docking conformation was further sampled through long-time scale (1 μs) molecular dynamics (MD) simulation to get the equilibrated binding states. Based on the simulation trajectory, per-residue binding free energy decomposition (Tu et al., 2018; Wang et al., 2019) and computational alanine scanning (CAS) (Huo et al., 2002) analysis were carried out to identify the protein–protein interface “hotspots.” Using one of the identified “hotspots” (Arg100) as an example, an additional 500 ns of MD simulation was performed to investigate the stability of the R100A mutant complex. Finally, the “hotspots” were mapped to the seven binding sites located at the OSM–OSMR interface detected using FTMap (Kozakov et al., 2011), and three of them were suggested as important target sites for future designs of small molecular modulators in the OSM–OSMR interaction.

## Materials and Methods

### Structure Preparation

#### Construction of OSM Missing Loop

The crystal structure and sequence of OSM were obtained from the PDB database (PDB code: 1EVS) (Deller et al., 2000). Residues from 1 to 3 and 135 to 155 (highlighted in red color in Table S1) were missing in the resolved crystal structure. The coordinates of the missing fragments of the OSM structure were constructed using the optimization-based approach (Fiser et al., 2000) in *Modeler* (Webb and Sali, 2016).

#### Homology Modeling of OSMR

The full-length sequence of the OSMR was obtained from the NCBI database (GenBank: AAI25210.1) (Strausberg et al., 2002). Then the sequence of the OSMR was submitted to search a template structure with the *BLAST* algorithm (Schaffer et al., 2001). Searching result showed that the sequence identity between the OSMR and LIFR was higher than 30%, especially in the protein-binding domain (57%). Therefore, based on the crystal structure (PDB code: 2Q7N) (Huyton et al., 2007) of the LIFR (residues from 201 to 383), 10 homology models of the OSMR protein-binding domain was constructed using *Modeler* (Webb and Sali, 2016).

### Protein–Protein Docking

OSM–OSMR docking was performed using the protein docking module of the latest version of Rosetta (Alford et al., 2017). Before docking, the PDB structures of OSM and OSMR were first formed through the script of *clean_pdb.py*. The formed structures of the two proteins were refined by running the Rosetta relax protocol, and the PDB files consisting of refined OSM and OSMR were generated. Then, according to the knowledge of the residues of OSM for OSMR binding detected by site mutagenesis studies (Adrian-Segarra et al., 2018b), the generated two complexes were loaded into *PyMOL* (Schrödinger, 2010) and with OSM reoriented to contact with the OSMR. To ensure low-energy starting side-chain conformations for docking, further prepacking of the OSM and OSMR complexes were conducted. Finally, 10,000 poses were calculated for the OSM–OSMR interactions using the Monte Carlo (MC) refinement method (Gray et al., 2003), with the pre-packed conformation as a starting point.

### Docking Funnel Analysis

With *InterfaceAnalyzer* mover in *RosettaScripts* (Fleishman et al., 2011), the RMSD was calculated from the heavy atoms of the interface residues (Table S2) using each pose of the top five scorers as a reference structure (Chaudhury et al., 2011). The docking funnel was then identified through plotting total_score against RMSD. Finally, the top scoring structure with the lowest RMSD was selected as the successful pose of the OSM–OSMR complex.

### Molecular Dynamics Simulation

Molecular dynamics (MD) simulation was performed with GPU-accelerated *PMEMD* in *AMBER14* (Babin et al., 2014). The selected near-native structure of OSM–OSMR from Rosetta docking was used as the initial conformation for MD simulation. The LEaP (Wang et al., 2006) was applied to assign *AMBERff14SB* force field parameters (Maier et al., 2015) for the two proteins, and two disulfide bonds in OSM and one disulfide bonds in the OSMR were identified and added. The complex was immersed into a rectangular periodic box of TIP3P (Hornak et al., 2006) water molecules, and the system was neutralized with two chloride ions. The distance between any protein atom and the edge of the box was set to 10 Å, and the prepared system contains 86,446 atoms per periodic cell. Starting from the representative snapshot of wild type OSM–OSMR, additional MD simulation was performed on the R100A complex using the same setup.

### MM/GBSA Binding Free Energy

The binding free energy (Δ*G*_tol_) between OSM and OSMR was estimated by the end-point molecular mechanics generalized Born surface area (MM/GBSA) approach (Kollman et al., 2000) as below:

(1) $$\begin{array}{r} \Delta G_{\text{tol}}=\Delta E_{\text{vdW}}+\Delta E_{\text{ele}}+\Delta G_{\text{pol}}+\Delta G_{\text{nonpol}} \end{array}$$

where Δ*E*_vdW_ and Δ*E*_ele_ are the van der Waals and electrostatic interaction energies, and Δ*G*_pol_ and Δ*G*_nonpol_ are the polar and non-polar solvent energies, respectively. Δ*E*_vdW_ and Δ*E*_ele_ were calculated using *AMBER ff14SB* (Maier et al., 2015) in the gas phase. Δ*G*_pol_ was calculated by solving the GB equation (Onufriev et al., 2004) with the dielectric constants of solute and solvent set to 1 and 80, respectively. Δ*G*_nonpol_ was calculated by Δ*G*_nonpol_ = γ × *SASA*, where γ = 0.0072, and *SASA* is referred to the solvent-accessible area and determined using a water probe radius of 1.4 Å (Sitkoff et al., 1994).

To further analyze the energy contribution between OSM and OSMR at a per-residue basis ($\Delta G_{\text{calc}}^{\text{per}-\text{residue}}$), the total binding free energy was decomposed by:

(2) $$\begin{array}{r} \Delta G_{calc}^{\text{per}-\text{residue}}=\Delta E_{\text{vdW}}^{\text{per}-\text{residue}}+\Delta E_{\text{ele}}^{\text{per}-\text{residue}}+\Delta G_{\text{pol}}^{\text{per}-\text{residue}}\\ \;+\Delta G_{\text{nonpol}}^{\text{per}-\text{residue}} \end{array}$$

The definition of each term in Equation (2) is similar as in Equation (1), except that *SASA* was computed by recursively approximating a sphere around an atom, starting from an icosahedron (ICOSA) (Babin et al., 2014).

### Computational Alanine Scanning Mutagenesis

Computational alanine scanning (CAS) mutagenesis was widely used to characterize the “hotspots” associated to protein–protein interactions (Huo et al., 2002). The whole process included the generation of mutated snapshots, and the binding free energy difference (Δ*ΔG*_calc_) between the wild type (WT) and mutant (MUT) complex is calculated below

(3) $$\begin{array}{r} \Delta \Delta G_{\text{calc}}=\Delta G_{\text{MUT}}-\Delta G_{\text{WT}} \end{array}$$

where *G*_WT_ and*G*_MUT_ refer to the MM/GBSA binding free energy of the WT and MUT complexes, respectively. Snapshot(s) of the WT of OSM-OSM and LIF-LIFR complex were collected from the last 500-ns trajectory and the crystal structure 2Q7N (Huyton et al., 2007), respectively. Alanine mutation was generated by truncating the selected mutation residue at Cγ and by replacing Cγ with a hydrogen atom at a 1.09-Å distance from Cß along the direction of the Cγ-Cß bond (Huo et al., 2002).

### Detection of Druggable Binding Sites

Based on the representative snapshot of the OSM–OSMR structure derived from the long-time simulation and the crystal structure of LIF–LIFR (Huyton et al., 2007), FTMap (Kozakov et al., 2011) was employed to detect the druggable binding site in the protein–protein interaction complexes. FTMap uses a fragment-based mapping algorithm that implements an efficient fast Fourier transform (FFT) correlation approach to search a global protein surface for potential druggable binding sites. The fragments include 16 small organic probe molecules (benzene, cyclohexane, ethane, ethanol, isopropanol, isobutanol, acetone, acetaldehyde, dimethyl ether, acetonitrile, urea, methylamine, phenol, benzaldehyde, acetamide, and N, N-dimethylformamide) of varying sizes, shapes, and polarities (Kozakov et al., 2015).

## Results and Discussion

### Modeled Structures of OSM and OSMR

The missing structures of OSM (Table S1), including the N-terminal fragment (1–3, AAI) and loop (135–155, SDTAEPTKAGRGASQPPTPTP), were built and refined using *Modeler* (Webb and Sali, 2016) because sequence identity between the loops of OSM and LIF (SKYHVGHVDVTYGPDTSGKDV) was only 10.3%. In addition, structural alignment indicated that the conformations of the two terminals that link the loops in the crystal structures of 1EVS and 2Q7N was significantly different (Figure S1). Therefore, the missing loop of OSM was predicted based on its own crystal structure 1EVS. Homology modeling approach in *Modeler* (Webb and Sali, 2016) was applied to provide the 3D structure of the OSMR binding domain (146–331) using the LIFR crystal structure (PDB code: 2Q7N) (Huyton et al., 2007) as a template. Figure 1B shows that the sequence identity between the OSMR and LIFR binding domain was 57%. As a result, 10 models were predicted for OSM and OSMR, respectively, and the model for each of them (Figures 1A,C) was selected by picking the structure with the best DOPE assessment score considering the Lennard–Jones potential and GBSA implicit solvent interaction (Shen and Sali, 2006).

### Prediction of OSM–OSMR Interaction Profiles

#### The Near-Native Conformation of OSM–OSMR Complex

To predict the OSM–OSMR binding funnel, RosettaDock was used to sample 10,000 poses from the starting position. The starting position was estimated according to the knowledge of binding site residues identified by site mutagenesis studies (Adrian-Segarra et al., 2018b), as the presence of a docking funnel is considered to be the most robust measure of success in a docking simulation (Chaudhury et al., 2011). Here, the top five scorers of the OSM–OSMR complex were used as references to plot the docking score of all 10,000 poses as a function of RMSD (Figure S2). One of the top five structures presenting the most reasonable docking funnel, in which the near-native conformations consistently have better scores than the non-native conformations (Chaudhury et al., 2011), is shown in Figure 1D. Therefore, the top scoring structures with the lowest RMSD in Figure 1D was selected as the initial conformation of OSM–OSMR for further studies. In addition, given that the structure of OSM is very similar to that of LIF, and the OSMR is modeled using the LIFR as the template, the structure of the OSM–OSMR complex was modeled based on the crystal structure of the LIF–LIFR complex. The calculated RMSD between the template-based and docking structures of the OSM–OSMR was 3.37 Å, suggesting that the two modeled structures are very similar with each other (Figure S3A). However, several spatial clashes were found between the interface of OSM and OSMR in the template-based OSM–OSMR complex (Figure S3B). As a result, it is proposed that the docking pose of the OSM–OSMR complex is more suitable for further investigation.

#### The Simulated Equilibration States of OSM–OSMR Complex

Starting from the docking conformation, 1 μs of all-atom MD simulation was performed for OSM–OSMR in explicit water. The time evolution of the RMSD of the Cα atom of proteins with respect to the initial coordinates of the docking pose is shown in Figure S4A. The RMSD values of OSM (~4 Å) and OSMR (~5 Å) showed that the two protein partners underwent conformation changes over the course of the simulation. In addition, compared with OSM and OSMR, the higher RMSD of the complex (~6 Å) suggested that the rotation of the two-partner orientation occurred. The extended root mean square fluctuation (RMSF) analysis of the protein residues indicated that the loop residues (135–155) in OSM were more flexible during the simulation; however, the interface residues in both OSM and OSMR were stabilized due to the non-bond interactions (Huang et al., 2019) between the two proteins (Figures S4B,C). Compared with the RMSF analysis of the OSM residues (Figure S4B) with the plot of B-factor of the LIF residues (12–180) in the crystal structure 2Q7N (Figure S5) indicated that OSM shares a similar structural fluctuation with LIF, especially in the loop region (135–155).

#### The Thermodynamics Properties of OSM–OSMR Complex

To characterize the thermodynamics properties between OSM and OSMR interaction, the snapshots derived from the last 500-ns equilibrated trajectory were used to estimate the MM/GBSA (Kollman et al., 2000) binding free energy. The decomposed energy terms of the total binding free energy (*G*_*tol*_) indicated that electrostatic interaction energy (*E*_*ele*_, −338.29 ± 44.73 kcal/mol), van der Waals interaction energy (*E*_*vdW*_, −82.02 ± 7.17 kcal/mol), and non-polar solvent energy (*G*_*nonpol*_, −11.74 ± 1.08 kcal/mol) play important roles in the formation of the protein–protein complex, whereas polar solvent energies (*G*_*polar*_, 383.11 ± 43.06 kcal/mol) were unfavored for the interaction.

**Figure 2:**
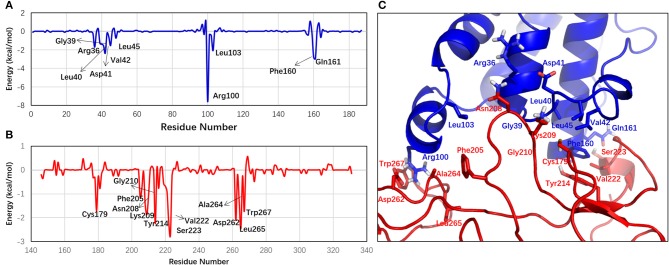
Per-residue energy profiles in **(A)** OSM and **(B)** OSMR contribute to the formation the complex. **(C)** The cartoon representation of the interaction mode of OSM–OSMR interface. Only the important residues (the absolute energy contribution ≥1 kcal/mol) are labeled.

In addition, per-residue energy decomposition analysis was performed to identify the important residues for the OSM–OSMR complex formation. The residues with an absolute energy contribution of more than 0.5 kcal/mol are listed in Table S3. The chart of the per-residue interaction energy and the interaction mode between OSM and OSMR are further shown in Figure 2. The per-residue energy decomposition analysis successfully predicted five residues in OSM (AB loop: Gly39, Leu40, Lys44, and Leu45; D helix: Phe160) reported by experiments, which played specific roles in activating OSMR signaling (Adrian-Segarra et al., 2018a,b). In addition, seven new residues (Arg36, Asp41, Val42, Arg100, Leu103, Gln161, and Leu164) in OSM were predicted as the important ones that contribute to the protein–protein interaction. Moreover, the 18 residues (Cys179, Leu181, Phe205, Ile206, Asn208, Lys209, Gly210, Tyr214, Glu216, Gln219, Gly220, Asn221, Val222, Ser223, Asp262, Ala264, Leu265, and Trp267) characterized in the OSMR were informative in experimentally verifying these residues, which may play an important role in OSM and OSMR interaction (Huang et al., 2019).

### “Hot Spots” Located at OSM–OSMR Interface

**Figure 3:**
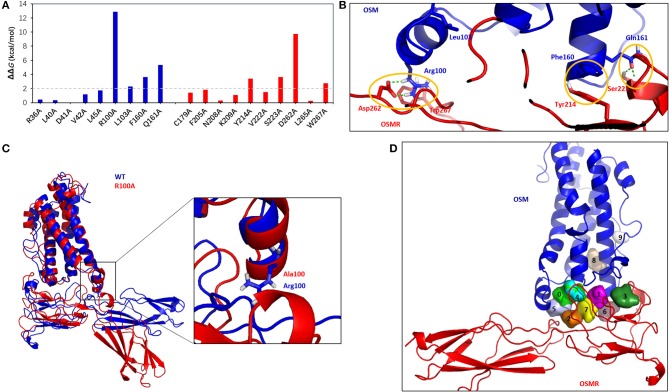
“Hot spots” and potential binding sites located at the OSM–OSMR interface. **(A)** Computational alanine scanning calculation of the 19 residues with absolute energy contribution of more than 1 kcal/mol identified in per-residue energy decomposition analysis. **(B)** Interactions of the eight “hot spots” located at the OSM–OSMR interface. The hydrogen bonds are displayed as green dashes. **(C)** Comparison of the equilibrated state conformation of wild-type OSM–OSMR with the snapshot of R100A mutant after 470-ns molecular dynamics (MD) simulation. **(D)** Potential binding sites in the OSM–OSMR complex identified through FTMap analysis. The detected 10 sites are labeled (0–9) and shown as surface with different colors in the structure.

In the context of protein–protein interaction, residues that made major contribution to the binding of free energy were termed as “hot spots,” which can be determined by alanine scanning mutagenesis (Zerbe et al., 2012). These “hot-spots” are highly interesting since the protein–protein interaction could be disrupted by targeting them (Grosdidier and Fernandez-Recio, 2008). Herein, to find the “hot spots” located at the OSM–OSMR interface, the computational alanine scanning (CAS) mutagenesis calculation was conducted on the residues with an absolute energy contribution of more than 1 kcal/mol identified by the per-residue energy decomposition analysis (Yang et al., 2018; Du et al., 2020). There were eight “hot spots” (OSM: Arg100, Leu103, Phe160, and Gln161; OSMR: Tyr214, Ser223, Asp262, and Trp267) with a relative binding free energy (*G*) of more than 2 kcal/mol (Figure 3A) (Moreira et al., 2007; Tu et al., 2018). Figure 3B clearly shows that some important non-bond interactions formed among those “hot spots,” such as the hydrogen bonds between Arg100 and Asp262, Gln161, and Ser223, and the π-π interaction between Phe160 and Tyr214. Among them, Phe160 was found to play an important role in OSM–OSMR recognition (Adrian-Segarra et al., 2018a,b). In addition, the other predicted “hot spots,” especially R100 and D262, were predicted to have a ΔΔG larger than 8 kcal/mol, which might be very useful for further theoretical and experimental studies. To investigate the stability of the mutant, using R100A complex as an example, an additional MD simulation (500 ns) was performed starting from the representative snapshot of wild-type OSM–OSMR. The calculated RMSD values of the OSM–OSMR complex are shown in Figure S4D. It is noted that RMSD significantly increased by around 470 ns for the R100A (~8 Å) complex. In addition, snapshots with the largest RMSD value during the simulation were extracted and shown in Figure 3C. Compared with the equilibrated state conformation of the wild-type OSM–OSMR, significant conformational change near the mutation site occurred in the R100A complex.

Moreover, using the crystal structure of the LIF–LIFR complex (PDB code 2Q7N), CAS analysis was performed on residues (Pro51, Phe52, Pro53, Leu56, Pro106, Leu109, Phe156, Gln157, Ile234, Val258, Asn261, Ser262, Ile267, Ile310, and Leu313) corresponding to the residues (Arg39, Asp41, Val42, Leu45, Arg100, Leu103, Phe160, Gln161, Phe205, Asn208, Lys209, Tyr214, Asp262, and Leu265) located at same position in the OSM–OSMR interface. It is found that Pro106, Phe156 in LIF, and Ile267 in LIFR (Figure S6), corresponding to Arg100, Phe160 in OSM, and Tyr214 in OSMR could be regarded as common “hot-spot” residues for both the OSM–OSMR and LIF-LIFR complexes. In the meanwhile, alanine mutations of other residues have little effect in the interaction energy of the LIF–LIFR complex, suggesting that the interface of the LIF–LIFR complex is significantly different from that of the predicted OSM–OSMR.

### Detection of Druggable Sites in OSM–OSMR Interface

Through FTMap (Kozakov et al., 2011) analysis of the MD simulation-derived structure of the OSM–OSMR complex, 10 potential druggable binding sites were detected from fragment-based searching of the global protein surface (Figure 3D), indicating that the conformation of the residues in the recognition interface is very flexible. To further verify the feasibility of the predicted OSM–OSMR model for potential binding sites analysis, the crystal structure of the LIF–LIFR complex was submitted for FTMap analysis using the same approach. The result showed that a total of 14 potential binding sites were detected, and the location of the position is similar with that of the OSM–OSMR complex (Figure S7). Therefore, it could be concluded that the predicted OSM–OSMR model is feasible for binding site prediction analysis. For the OSM–OSMR complex, the protein residues that interacted with bound fragments (within 4 Å) in the binding sites are summarized in Table 1. As shown in Table 1, six of the 10 sites (sites 0, 1, 3–6) were located at the interface of the OSM and OSMR interaction. Sites 2 and 7 were located in the OSMR (Table 1), and sites 8 and 9 were found in OSM (Table 1 and Figure 3D).

**Table 1:** List of key interacting residues within 4 Å of the bound probe molecules in the detected potential binding sites rendered as spheres in Figure 3D.

**Site**	**OSM**	**OSMR**
0	Arg36, Ile37, Gln38, Gly39, Pro93, Asp97, Leu98, Ser101, **Leu103**	Ile206, Arg207, Asn208, Lys209
1	Gln38, Gly39, Leu40, Leu45, **Phe160**, Lys163	Ser178, Cys179, Gly210, Thr211, Asn212, **Tyr214**,
2		Phe205, Ile206, Leu231, Phe232, Val233, Ser234, Ala264, Leu265, Gly266
3	Lys44, Leu45, His48, **Phe160**	Asn176, Val177, Ser178, **Tyr214**, Cys215, Glu216, Ser218, Gln219, Gly220, Val222
4	Arg36, Ile37, Gly39, **Leu103**	Ile206, Arg207, Asn208, Lys209, Gly210
5	Asp97, Leu98, **Arg100**, Ser101	Ile206, Ala264, Leu265, Gly266
6	Asp158, Ala159, **Phe160**	Gln146, Asn212, **Tyr214**, Val222, Lys227, Gly228, Val230
7		Ile206, Gly210, Thr211, Asn212, Leu231, Val233
8	Arg84, Asp87, Leu88, Arg91, Arg162, Glu165, Gly166	
9	Arg84, Pro151, Thr152, Pro153	

*The identified “hot spots” by computational alanine mutagenesis were shown in bold*.

To further evaluate the druggability of the detected binding sites in the OSM–OSMR complex, the identified eight “hot spots” were mapped to the protein residues summarized in Table 1. Interestingly, two common “hot spots” (OSM: Phe160, OSMR: Tyr214) were found in sites 1, 3, and 6. However, only one hot spot (OSM: Arg100 or Leu103) was found in sites 0, 4, and 5, and no “hot spot” was found in sites 2, 7, 8, and 9. This could be understand through the relationship between “hot spots” and ligand binding “hot spots” in the protein–protein interface, in which additional topological requirements were needed in a “hot spot” for small molecule binding (Zerbe et al., 2012). Therefore, sites 1, 3, and 6 were important target sites for designing inhibitors that may inhibit the protein–protein interaction between OSM and OSMR. In addition, as the binding site analysis was performed on the global protein surface, the predicted sites 2, 7 in OSMR and sites 8, 9 in OSM, especially the latter two sites (Figure 3D), which are located far away from the interface, could be regarded as potential allosteric sites.

## Conclusion

Targeting the OSM and OSMR pathway represents a potential strategy for the treatment of IBD. In this work, the interaction between OSM and OSMR was investigated by employing computational simulation techniques including homology modeling, protein–protein docking, and long-time scale MD simulation. Post-analysis of the equilibrated simulation trajectory characterized seven new residues in OSM and 18 residues characterized in the OSMR as the important ones contributing to the protein–protein interaction. Based on these important residues, computational alanine scanning and FTMap analysis detected eight “hot spots” and six potential binding sites located at the OSM–OSMR interface. It is interesting to note that, compared with the equilibrated state conformation, significant conformational change near the mutation site occurred in the R100A (one of the identified “hot spots”) complex during MD simulation. Further mapping of the eight “hot spots” in the detected binding sites suggested that sites 1, 3, and 6 were important target sites, which may be used for designing inhibitors to block OSM and OSMR interaction.

## Data Availability Statement

All datasets generated for this study are included in the article/Supplementary Material.

## Author Contributions

QD and WX designed the experiments and performed computational simulations. QD, YQ, and WX analyzed the data and wrote the paper.

### Conflict of Interest

The authors declare that the research was conducted in the absence of any commercial or financial relationships that could be construed as a potential conflict of interest.
