# Supplementary material for: Molecular Simulation of Oncostatin M and Receptor (OSM–OSMR) Interaction as a Potential Therapeutic Target for Inflammatory Bowel Disease
Source: Front Mol Biosci. 2020 Mar 4;7:29. doi: 10.3389/fmolb.2020.00029 (PMC7064634; doi:10.3389/fmolb.2020.00029)
Supplement: Supplementary file 1 [file Data_Sheet_1.pdf]

Supplementary Material For

Molecular Simulation of Oncostatin M and Receptor (OSM-OSMR) Interaction as a Potential Therapeutic Target for Inflammatory Bowel Disease

Qingqing Du[1], Yan Qian[1]*, Weiwei Xue[2]*

[1] Depart of Pharmacy, The Second Affiliated Hospital of Chongqing Medical University, Chongqing 400010, China

[2] School of Pharmaceutical Sciences, Chongqing Key Laboratory of Natural Product Synthesis and Drug Research, Chongqing University, Chongqing 401331, China

***Corresponding Author**

Dr. Weiwei Xue

E-mail: xueww@cqu.edu.cn

Phone: +86-(0)23-6567-8468

Dr. Yan Qian

E-mail: cqqianyan@hospital.cqmu.edu.cn

Phone: +86-(0)23-6369-3137

Table S1. The sequence information of proteins used in this work, the missing fragments of PDB structure 1EVS (1) was highlighted in red color.

>PDB database: 1EVS, Chain A Oncostatin M 1-187 [Homo sapiens]
AAIGSCSKEYRVLLGQLQKQTDLMQDTSRLLDPYIRIQGLDVPKLREHCRERPGAFPSEE TLRGLGRRGFLQTLNATLGCVLHRLADLEQRLPKAQDLERSGLNIEDLEKLQMARPNIL GLRNNIYCMAQLLDNSDTAEPTKAGRGASQPPTPTPASDAFQRKLEGCRFLHGYHRFMH SVGRVFSKW
> GenBank: AAI25210.1, Oncostatin M receptor 146-331 [Homo sapiens]
QDILFVFPKDKLVEEGTNVTICYVSRNIQNNVSCYLEGKQIHGEQLDPHVTAFNLNSVPFI RNKGTNIYCEASQGNVSEGMKGIVLFVSKVLEEPKDFSCETEDFKTLHCTWDPGTDTAL GWSKQPSQSYTLFESFSGEKKLCTHKNWCNWQITQDSQETYNFTLIAENYLRKRSVNILF NLTHRV

Table S2. OSM-OSMR interface residues used to RMSD calculation in docking funnel analysis.

OSM	OSMR
Gln38, Cys49, Leu92, Ile105, Pro155, Arg162	Gln188, Asp197, Val219, Glu224, Phe247, Ser276, Asp304, Gln312, Cys330, Asn334

Table S3. Energy contributions of the identified hot spots residues located at the interaction interface of the protein-protein complexes

OSM		OSMR	
Residues	Energies	Residues	Energies
**Arg36**	**-1.71**	**Cys179**	**-1.71**
**Gly39**	**-1.32**	Leu181	-0.78
**Leu40**	**-1.39**	**Phe205**	**-1.14**
**Asp41**	**-1.56**	Ile206	-0.77
**Val42**	**-2.36**	**Asn208**	**-1.47**
Lys44	-0.6	**Lys209**	**-1.87**
**Leu45**	**-1.54**	**Gly210**	**-1.05**
His48	-0.74	**Tyr214**	**-2.25**
**Arg100**	**-7.61**	Glu216	-0.77
**Leu103**	**-2.1**	Gln219	-0.58
**Phe160**	**-2.82**	Gly220	-0.68
**Gln161**	**-2.97**	Asn221	-0.91
Leu164	-0.66	**Val222**	**-2.18**
		**Ser223**	**-2.73**
		**Asp262**	**-2.11**
		**Ala264**	**-1.32**
		**Leu265**	**-2.44**
		**Trp267**	**-1.74**

[a] Per-residue binding free energies decomposed using MM/GBSA method (2).

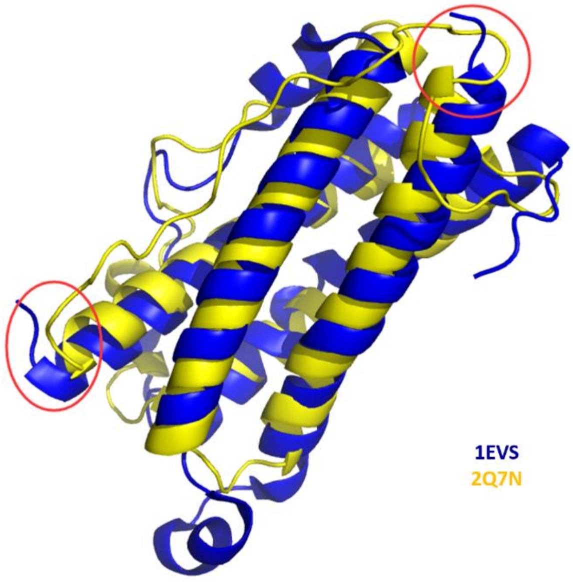

Figure S1. Structural alignment of the crystal structures of OSM (1EVS) and LIF (2Q7N).

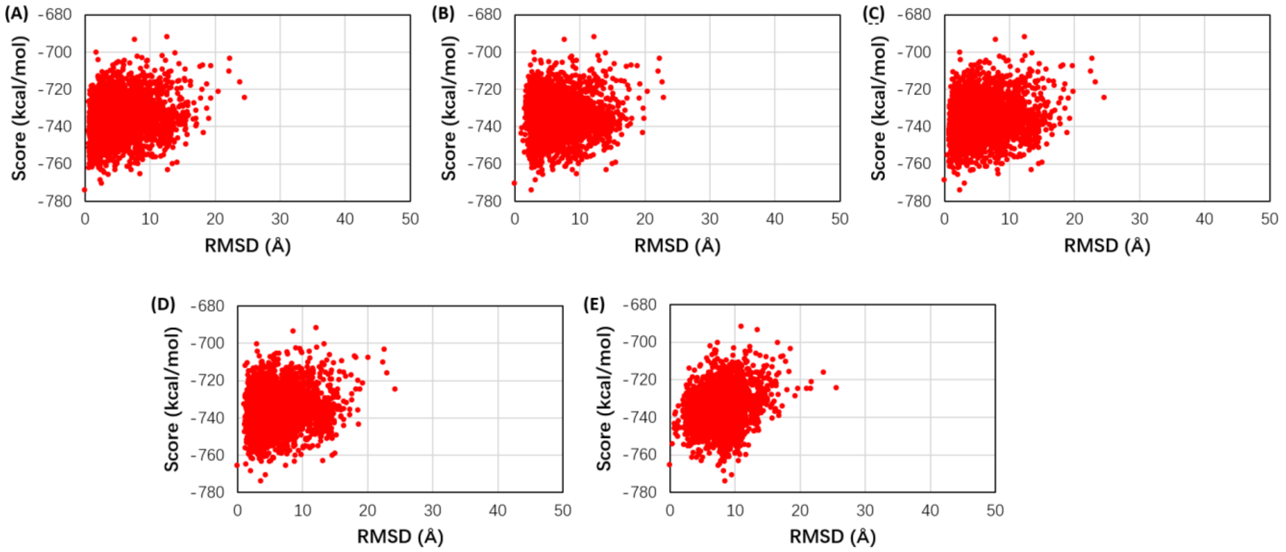

Figure S2. Docking score vs interface RMSD scatter plots for using the top five docking poses (A-E) as a reference structure, respectively.

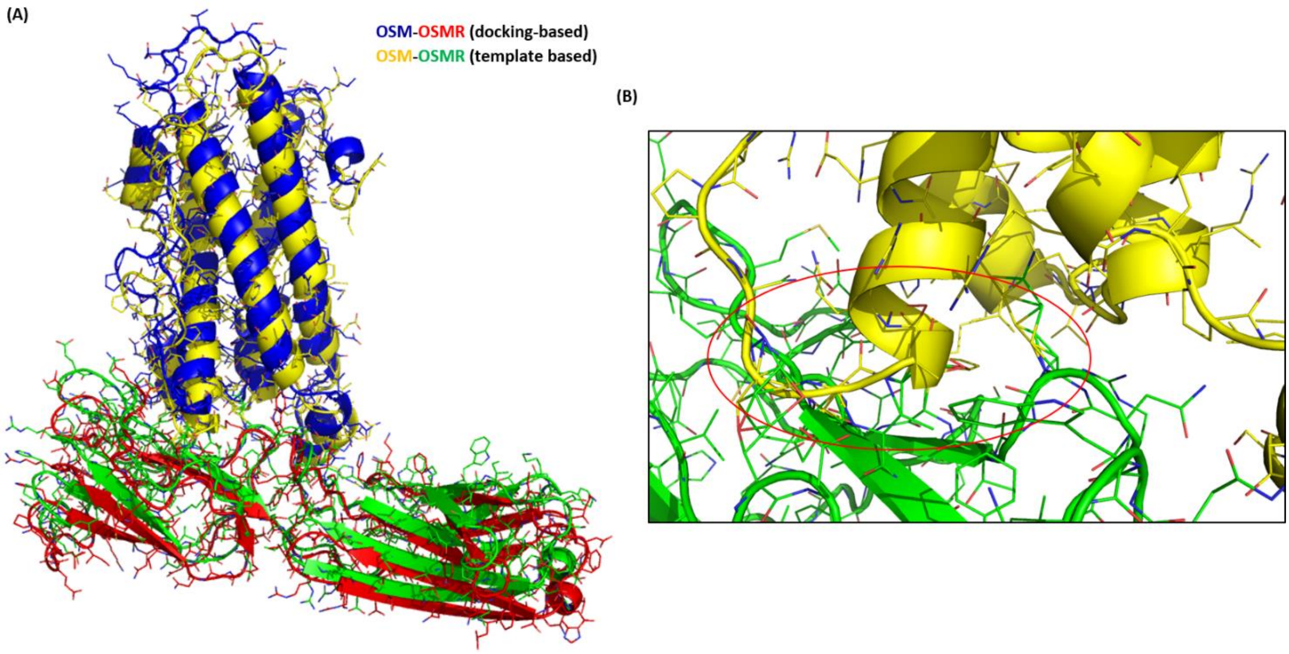

Figure S3. Comparison of the structures of OSM-OSMR complex based on the crystal structure of the LIF-LIFR complex and docking.

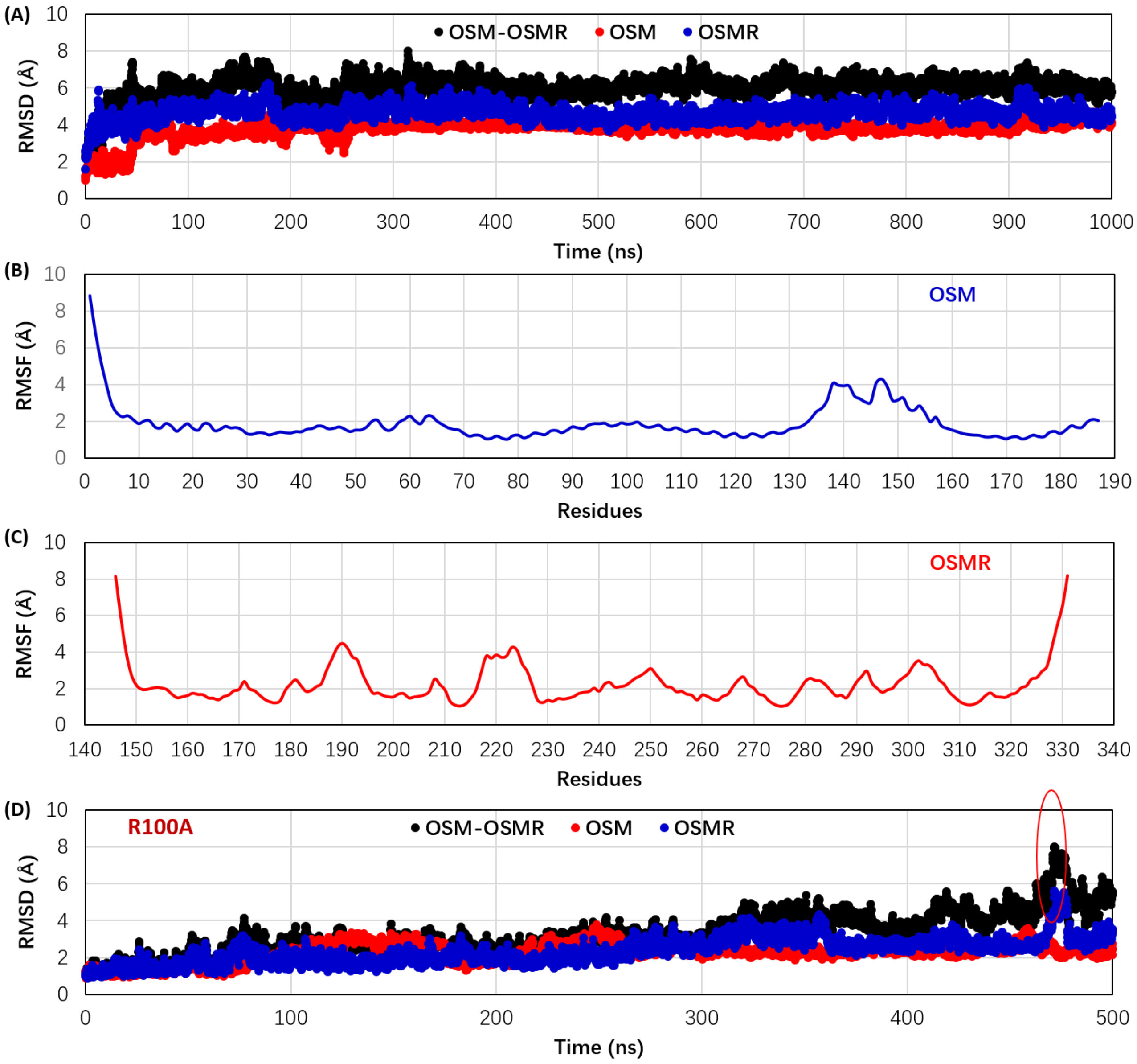

Figure S4. (A) The time evolution of the root-mean-square deviation (RMSD) of Cα atom of proteins with respective to the initial coordinates of the docking pose. (B and C) The root mean-square fluctuations (RMSF) analysis of OSM and OSMR during MD simulations. (D) The time evolution of the RMSD values of Cα atom of proteins in OSM-OSMR R100A complex with respective to the equilibrated wild type conformation.

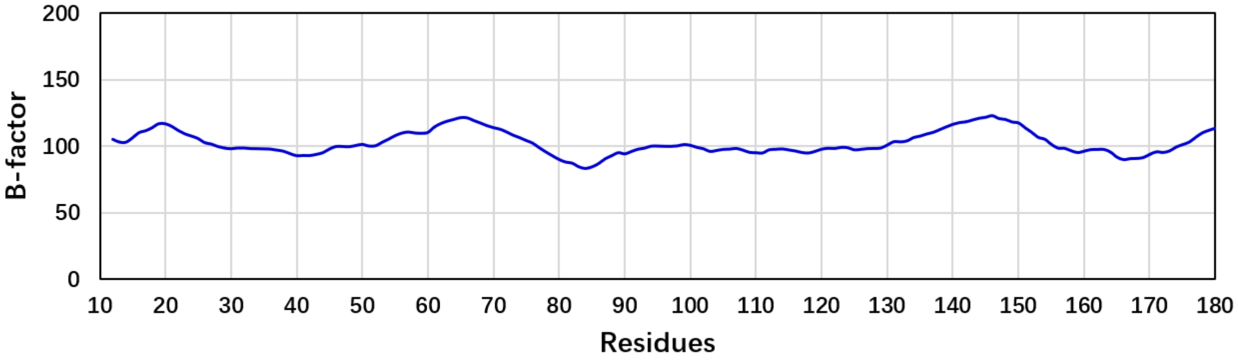

Figure S5. Plot of B-factor of LIF residues (12-180) in crystal structure 2Q7N.

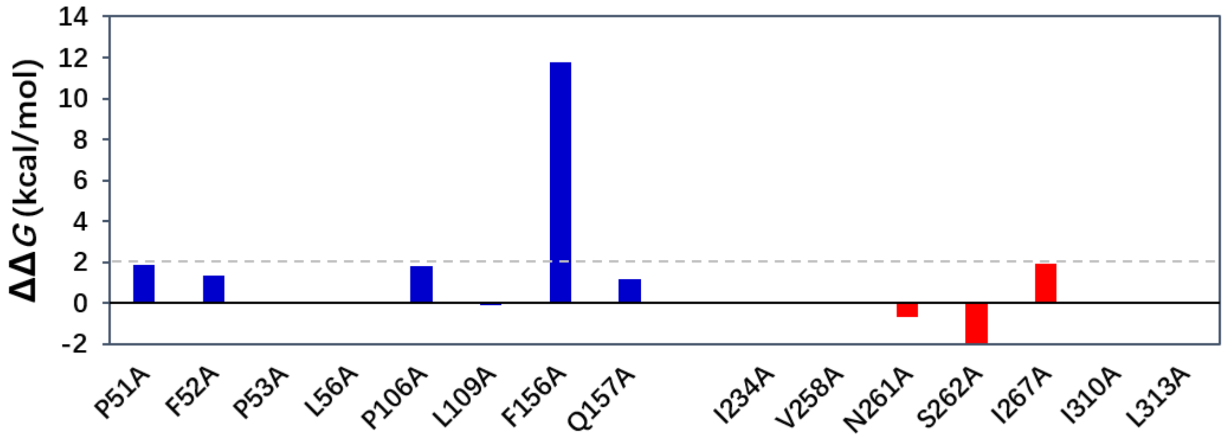

Figure S6. Computational alanine scanning analysis of LIF-LIFR complex residues corresponding to the residues located at same position in OSM-OSMR interface.

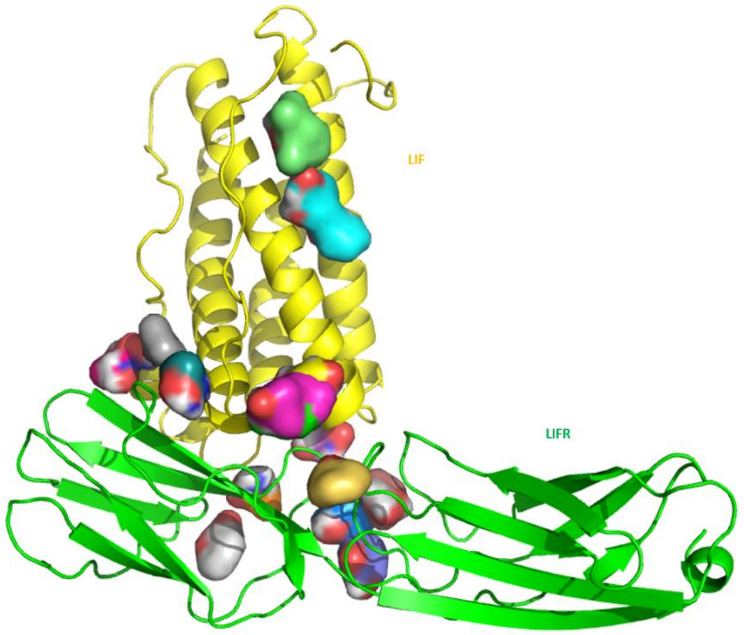

Figure S7. Potential binding sites in the LIF-LIFR complex identified through FTMap (3) analysis.

References

1. M. C. Deller, K. R. Hudson, S. Ikemizu, J. Bravo, E. Y. Jones and J. K. Heath: Crystal structure and functional dissection of the cytostatic cytokine oncostatin M. *Structure*, 8(8), 863-74 (2000) doi:10.1016/s0969-2126(00)00176-3

2. P. A. Kollman, I. Massova, C. Reyes, B. Kuhn, S. Huo, L. Chong, M. Lee, T. Lee, Y. Duan, W. Wang, O. Donini, P. Cieplak, J. Srinivasan, D. A. Case and T. E. Cheatham, 3rd: Calculating structures and free energies of complex molecules: combining molecular mechanics and continuum models. *Acc Chem Res*, 33(12), 889-97 (2000) doi:10.1021/ar000033j

3. D. Kozakov, D. R. Hall, G. Y. Chuang, R. Cencic, R. Brenke, L. E. Grove, D. Beglov, J. Pelletier, A. Whitty and S. Vajda: Structural conservation of druggable hot spots in protein-protein interfaces. *Proc Natl Acad Sci U S A*, 108(33), 13528-33 (2011) doi:10.1073/pnas.1101835108
